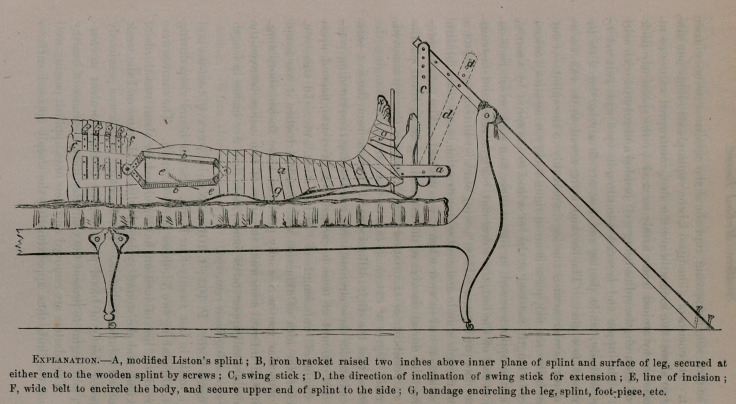# Excision of the Upper Extremity of the Femur, after Compound Comminuted Fracture

**Published:** 1875-05

**Authors:** P. A. Harris

**Affiliations:** Dover, New Jersey


Vol. V.—MAY, 1875.—No. 5.

### Original Communications.

### EXCISION OF THE UPPER EXTREMITY OF THE FEMUR, AFTER COMPOUND COMMINUTED FRA C TURE.

BY P. A. HARRIS, M. D., DOVER, NEW JERSEY.

August 23, 1873, J. P., æt. 29, single, while engaged in iron-mining was crushed by a falling mass of rock weighing 300 pounds, producing a compound comminuted fracture of the upper one-fourth of the femur, including trochanter and a portion of neck. An examination revealed the fact that the wound on the anterior aspect of the thigh, situated just at the outer and lower border of Scarpa's triangle, was large enough to admit the hand. The bony fragments were widely separated, but the larger blood-vessels and nerves remained intact.

After a further careful examination, assisted by Drs. Condict and Richie, called in consultation, it was decided to perform the operation of excision, remove all fragments, saw off the sharp, projecting portions of the obliquely fractured upper end of lower bone, and place our patient in the best possible condition for recovery. Four hours subsequent to the injury, after thorough anæsthesia, the operation was begun by introducing in the wound a curved director carrying a chain-saw, which was carried round the axis of the upper end of lower fragment, and the sharp oblique extremity sawn transversely across. The patient being then placed on his left side, we introduced the knife at a point midway between the anterior superior spinous process and trochanter major, cutting slightly backwards, then downward and forward, forming a convex incision with the concavity looking forward, according to the plan pursued by most surgeons for hip-joint excision. This exposed to view the fractured neck and trochanter, and all the fragments, which were removed, together with the head of the bone. The wound was then thoroughly sponged out and closed immediately by sutures, between which were placed long strips of adhesive plaster, thus securing perfect coaptation, except the most dependent portion of the incision, which was left open for drainage. The operation being completed, the patient was laid on a level bed, and the leg bandaged to an ordinary double-inclined plane until a permanent apparatus could be devised for keeping the limb in a desired position. Returning in the evening, found him comfortable and quite free from pain.—Ordered beef-tea and liq. morph. sul. ʒi. every hour till sleep was produced.

August 24, 9 A. M., p. 107; t. 101°. Patient complains of soreness at site of the operation. Four hours sleep during night; has taken ℥xvi. beef-tea since yesterday. Ordered ℥iij. beef-tea every two hours and the wound to be syringed out with tepid water, followed by a weak solution of carbolic acid. Returned home, and with the assistance of a blacksmith constructed the following apparatus, which Dr. Condict assisted me in fitting to our patient twenty-four hours subsequent to the operation:

EXPLANATION.—A, modified Liston's splint; B, iron bracket raised two inches above inner plane of splint and surface of leg, secured at either end to the wooden splint by screws; C, swing stick; D, the direction of inclination of swing stick for extension; E, line of incision; F, wide belt to encircle the body, and secure upper end of splint to the side; G, bandage encircling the leg, splint, foot-piece, etc.

The foregoing engraving represents the appliance after adjustment. It consists of a modified Liston's splint, extending from the body to about five inches below the foot, interrupted at the point of incision by arched irons firmly secured to the splint on either side of the arch, the splint being well padded and covered with canvass. The upper end is secured to the side by a wide belt encircling the body, while the leg and foot are firmly bandaged to the splint and foot-piece. The bandage being sewed to the canvass at the upper border secures the leg immovably to the splint. Four inches below the foot-piece is an upright stick, (swing stick), fifteen inches in length, fastened at the lower end to the splint by a screw, and at the upper screwed in like manner to a stick which rests on the foot-rail of the bed, the lower end being beveled and fastened to the floor by screws. This "swing stick" addition to Liston's splint, though simple and easy of construction, more than fulfilled the indication had in view when it was devised. The patient, preserving the dorsal decubitus, the foot and leg being lifted off the bed, all possibility of excoriation from pressure is thereby removed. A certain degree of extension can be maintained by changing the screws so that the stick inclines several degrees from the foot—the leg itself, under these circumstances, constituting the extending power. The amount of tension exerted will, of course, depend upon the weight of limb, length of "swing stick," etc. In this case, however, the idea of extension was abandoned when it was found that muscular contraction only produced three inches shortening, leaving an interval somewhat in excess of two inches between the acetabulum and upper end of femur, more than five inches in length of bone having been removed.

August 25th. P. 103; t. 101°. Nearly ℥ xxxvij. beef-tea and ℥ viij. milk in last twenty-four hours. Seven hours sleep during night, after taking two ʒ i. doses liq. mor. sulph. The sutures were removed, the line of incision having closed for about two-thirds of its extent, and the wound syringed with tepid water and the weak carbolized solution as before. The discharge, which was previously composed chiefly of blood products, has become purulent in character, and begins to present the characteristic appearance of healthy pus. The plasters are removed and new ones applied, passing well around the limb, thus approximating the deeply opposed surfaces of the incision. Patient quite free from pain, and the wound presents a healthy appearance.

From this date, until September 6, the wound was daily cleansed with the tepid water and solut. carb. acid ; the wet and loosened adhesive straps were replaced by new ones from day to day, and the belt encircling the body, securing the splint to the side, was made tight or loosened as the comfort of the patient seemed to demand, while the bandage securing the splint to the leg was entirely removed every six or seven days, and a new one applied, which, after adjustment, was sewed to the upper border of the canvass covering the splint. Patient consumed one to two pints of beef-tea and one pint of milk every twenty-four hours—no tonics or stimulants being given—and averaged nightly from six to eight hours' sleep.

September 6. But little pain ; rests well, morphia being dispensed with part of the time ; appetite good, without tonics, though pulse is not quite so strong. Discharge abundant, having, in general, a laudable look. Heretofore, in syringing, the long vaginal nozzle has been introduced in the wound easily every day. Now it passes with so much difficulty that we are compelled to substitute a smaller one, affording substantial evidence of the rapidly healing process Nature has instituted to supply the deficiency. The syringe enters to the extent of four or five inches, discharging its contents in the region of the acetabulum, and cleaning the parts completely. Pulse being weak, ℥ ij. of brandy are ordered every twenty-four hours.

September 11. Doing well. Wound will not admit nozzle of smallest syringe ; consequently injections are made tnrough the incision. When this is done, a few drops of the injected fluid find exit from the wound. The walls of the cavity are, doubtless, all reached by the injections.

September 25. No important change in patient's general condition. Occasionally the discharge loses its opaque, yellow appearance, and becomes thin and colorless, irritating the parts with which it comes in contact. This condition, however, is transient, soon giving place to healthy pus. The wound is rapidly filling with granulations, the diameter being gradually diminished by the contracting cicatricial tissue.

October 14. Patient looking well. Very little pain ; sleeps well; appetite good; wound entirely healed. The deep portions of the incision appear, for the most part, to be well united. One or two sinuses leading to the region of the acetabulum continue to discharge a small quantity, perhaps an ounce, during the twenty-four hours. Decided, to-day, to remove the long splint, and, Dr. Condict assisting, substituted a plaster of Paris bandage. In it were laid long pine splints, about one inch wide and $\frac{1}{16}$ to $\frac{1}{8}$ inch thick, this addition adding very largely to its strength as a dressing. A fenestrated opening at the point of incision admits easy dressing of the wound as before. Bandage comfortable.

October 23. Assisted patient out of bed and had him stand, aided by crutches, the injured limb being supported by a rubber sling, extending from the foot to the neck. This is made of heavy, half-inch rubber tubing, and, without tension, reaches to the calf of the leg, so that a weight equal to that of the limb is required to stretch it to the foot, under which it passes. An elastic sling in a case of this kind appears to accomplish what nothing else can, i. e. to give almost equal support to the leg in all the varied positions assumed by the patient.

October 24. Stands, and, assisted, can walk with crutches.

October 25. Remains out of bed fifteen minutes, walking well on crutches, having some one to steady him. Direct him to leave bed every day and remain up long as possible.

November 15. Doing well; discharge diminishing; has gained strength rapidly since getting out of bed; remains up two or three hours each day. Plaster of Paris bandage removed; the leg retains its acquired position and is quite firmly united at the hip.—Patient has slight power of flexion and extension. No reasonable force causes any perceptible difference in the length of the limb.—One sinus still discharges small quantity healthy pus.

December 15. Patient doing finely; up all day; can bear half his entire weight on the injured limb.

At date of this writing—January 17—almost six months have elapsed since the injury, and the case seems sufficiently advanced to warrant us in giving a prognosis. One sinus still discharges slightly, yet the patient can bear his whole weight on the injured limb without any pain whatever; looks well, and has a fair appetite.—Direct him to take plenty of exercise without using the injured leg, hoping the sinus and lesion to which it leads may entirely heal.—Careful probing fails to afford any evidence of necrosed bone.

Over five inches of the longitudinal axis were removed. Shortening exists to the extent of three inches. This can be compensated by a cork or steel extension-shoe. Judging from his present condition, it will not be long before he begins to use the injured member, which, though short, will be infinitely more serviceable than any artificial limb, adjusted after a hip-joint amputation. In view of these facts, we regard the operation as successful, not only in saving the life of our patient, but in sparing him a limb which will be of great service in locomotion. The operation of excision of the head, neck and trochanters of the femur is not, in itself, very dangerous, as is proved by the fact that forty-three per cent. of all operations made for the removal of necrosed bone end in recovery. It must be remembered, however, that the great majority of cases operated on for chronic diseases are children; who possess a high degree of vitality, ninety per cent. at least, not having attained the age of sixteen years, enabling a larger proportion of them to recover than adults.

Where excision has been performed for chronic disease, in adults of thirty years and upward, only fifteen per cent. have recovered. With this knowledge of the mortality following the operation, we are still unable to draw the line of difference and determine how much of the danger in question depends on the knife, or how much is added by the disease which existed months or years before.

There are eighty-five recorded cases of excision of head, neck and trochanters, after injury, with eight recoveries. Twelve cases are reported prior to our late war, of which only one recovered. Of the eighty-five cases, seventy-three occurred in military practice, from which it is safe to infer they were mostly after gun-shot injury. Hence, how seldom has the operation been resorted to in private practice.

If I may add to this number the case in question, we have a total of eighty-six operations, with nine recoveries, or more than ten per cent. of the cases ending favorably. The results obtained after hip-joint amputations in military practice show that *less* than ten per cent. recover; so that excision may be considered less fatal, while it offers to those who recover an impaired though, in most cases, useful limb.

Another plan—which may well be called "expectant"—of treating this class of injuries, is to reduce the fractured bone as far as possible, and treat as a case of compound fracture, simply.

So far as we can learn, this has been much less satisfactory than excision or amputation; so that, in a majority of the cases of compound comminuted fracture of the femur, where the fragments are widely separated, we are left only to consider the relative value of the two latter operations.

If the wound be not too extensive, and the femoral vessels and nerves remain intact, it is a case for excision. When the reverse obtains, it is probable the only hope of a successful issue lies in amputation. May it not be laid down as a rule in surgery, that excision should, in all cases, be preferred to amputation where the large blood-vessels and nerves are uninjured?

In exsection it is, doubtless, best to remove as little as possible, viz:

All separated fragments;

All the sharp projecting portions of the bone;

The head and neck, if the capsular ligament or any of the intra-capsular portion of the bone is found to be injured.

If the fracture involves the trochanters and only that portion of the neck without the capsular ligament, it will doubtless be best to saw off the neck just external thereto, removing the injured portion and leaving the head intact. This, in general, cannot be decided upon until the incision is made, through which to examine the parts.

The fearful rate of mortality attending amputation at the hip-joint, and also the "expectant" plan of treatment, have been as amply demonstrated in civil as in military practice. Most of the cases of excision after injury, have occurred in military service, where the mortality attending capital operations is much greater. In view of these facts, it may safely be inferred that in non-military surgery, with a proper selection of cases, the operation promises more than we have seen from it in the past.

---

Deaths from London Fog.—According to the *Lancet*, ten inquests were held November 24th on the bodies of persons whose death was attributed to the effects of the fog.

## Figures and Tables

**Figure f1:**